# Detection of Structural Changes Prior to the Burst of a Hydrogen Composite Overwrapped Pressure Vessel Using Ultrasonic Guided Waves

**DOI:** 10.3390/s26175589

**Published:** 2026-09-03

**Authors:** Houssam El Moutaouakil, Jan Heimann, Daniel Lozano, Enes Savli, Jens Prager, Andreas Schütze

**Affiliations:** 1 Lab for Measurement Technology, Saarland University, 66123 Saarbruecken, Germany; schuetze@lmt.uni-saarland.de; 2 Federal Institute for Materials Research and Testing, 12205 Berlin, Germany; jan.heinmann@bam.de (J.H.); daniel-hernando.lozano-duarte@bam.de (D.L.); jens.prager@bam.de (J.P.); 3 Fraunhofer Institute for Ceramic Technologies and Systems (IKTS), 01109 Dresden, Germany; enes.savli@ikts.fraunhofer.de

**Keywords:** ultrasonic guided waves, structural health monitoring, burst detection, structural damage, damage detection, composite overwrapped pressure vessel, interpretable machine learning

## Abstract

**Highlights:**

**What are the main findings?**

- Up to 98.28% balanced accuracy was achieved for distinguishing baseline and post-transition states.
- A persistent structural transition was detected before burst failure and remained detectable after unloading the vessel.

**What are the implications of the main findings?**

- Interpretable Best Daubechies Wavelet features are combined with k-Nearest Neighbors novelty detection.
- The model was validated using experimental ultrasonic guided wave measurements from a real-world composite pressure vessel.

**Abstract:**

Composite overwrapped pressure vessels are increasingly used for hydrogen storage because of their lightweight construction. Ensuring their structural integrity therefore becomes an important requirement for safe operation. Ultrasonic guided waves are well suited for this task because they are highly sensitive to structural changes in thin-walled pressure vessels. In this work, we developed a machine learning framework based on interpretable Best Daubechies Wavelet features and k-Nearest Neighbors novelty detection. The framework identifies a persistent transition in the UGW response during overpressurization that is indicative of a permanent structural change and occurs prior to burst failure. It was validated using measurements acquired from a real-world pressure vessel. For the a priori selected sensor pair 11–12, located in the highly stressed cylindrical section, the method achieved a balanced accuracy of 98.28% and a true negative rate of 100%. In addition, the proposed methodology identified the pressure level at which the persistent structural transition first became detectable and showed that this transition remained detectable after the vessel had returned to its normal operating pressure.

## 1. Introduction

Composite overwrapped pressure vessels (COPVs) are gaining an increasing role in hydrogen storage as a green energy carrier [1]. Furthermore, the lightweight properties of COPVs make them attractive for applications where weight is critical, such as in airspace applications [2]. Due to the high-explosive nature of hydrogen gas and its storage under high pressure, the safety of these pressure vessels needs to be ensured [3]. For this purpose, various structural health monitoring (SHM) methods exist that offer real-time non-destructive testing (NDT) [4].

One powerful inspection method employs ultrasonic guided waves (UGWs) [5]. This type of ultrasonic wave is usually operated using the pitch-catch procedure and propagates long distances with minimal energy loss [6,7]. Additionally, it is sensitive to structural changes that can lead to failure [8]. Those failure modes, such as matrix cracking, delamination, and fiber breakage, are inherent to the COPV’s anisotropic, multi-layered construction [9,10]. Given the high pressure involved, real-time continuous monitoring is essential to ensure operational safety and prevent catastrophic burst failure [11].

However, applying UGWs in practice is challenging because of signal dispersion, wave-mode overlap, and environmental noise [12,13,14]. To handle these complex signals and automate their evaluation, research now focuses on machine learning (ML) [15,16,17]. Supervised models work well in laboratories but require large datasets with multiple samples of failure events, which are typically unavailable for real-world infrastructure [18]. Additionally, standard validation methods such as random data splitting often lead to overly optimistic results due to data leakage between similar pressure cycles [19]. Related developments in adjacent fields include guided-wave-based SHM of composite structures and other safety-critical components, where similar challenges arise regarding environmental variability, limited damage data, and reliable feature extraction [5,16].

To address these issues, this paper proposes a semi-unsupervised novelty detection pipeline. Novelty detection enables models to be trained exclusively on measurements acquired in the healthy state and to identify deviations from this reference state [20]. The employed method extracts features from UGW signals that characterize changes in their time-frequency content and subsequently uses these features to identify deviations from the baseline state. The underlying framework for automated feature extraction and selection in condition monitoring is described in [21]. We trained a k-Nearest Neighbors (kNN) algorithm [22] only on baseline data, thereby avoiding measurements acquired after overpressurization and focusing on detecting deviations from the pristine state. We used a group-based validation method, where each group represents an independently conducted pressure ramp, to prevent data leakage between measurements acquired within the same experimental sequence. Specifically, Leave-One-Group-Out Cross-Validation (LOGO-CV) was applied [23]. By inspecting the extracted features and the resulting novelty scores, we identified the onset of persistent changes in the UGW response. Detecting this transition provides an early indication of persistent structural changes before burst failure.

The individual processing and machine learning methods used in this framework are established techniques. The methodological contribution of this study lies in using a novelty detection formulation that requires only measurements representing the healthy structural state for training and applies it to the detection of persistent structural changes during progressive pressurization of a COPV. To the best of our knowledge, no previous study has demonstrated healthy-state-only novelty detection using ultrasonic guided waves for identifying a persistent pre-burst structural transition in a COPV. In contrast to supervised classification or regression approaches, measurements representing structural degradation or damage are not required during model training.

## 2. Design of Experiments

The experimental campaign was performed using a high-pressure burst testing system (Dustec Hochdrucktechnik GmbH, Wismar, Germany), which allows operation in both pressure- and volume-controlled modes with a maximum pressure of 2300 bar. In the present study, all tests were conducted under pressure-controlled conditions.

**Figure 1:**
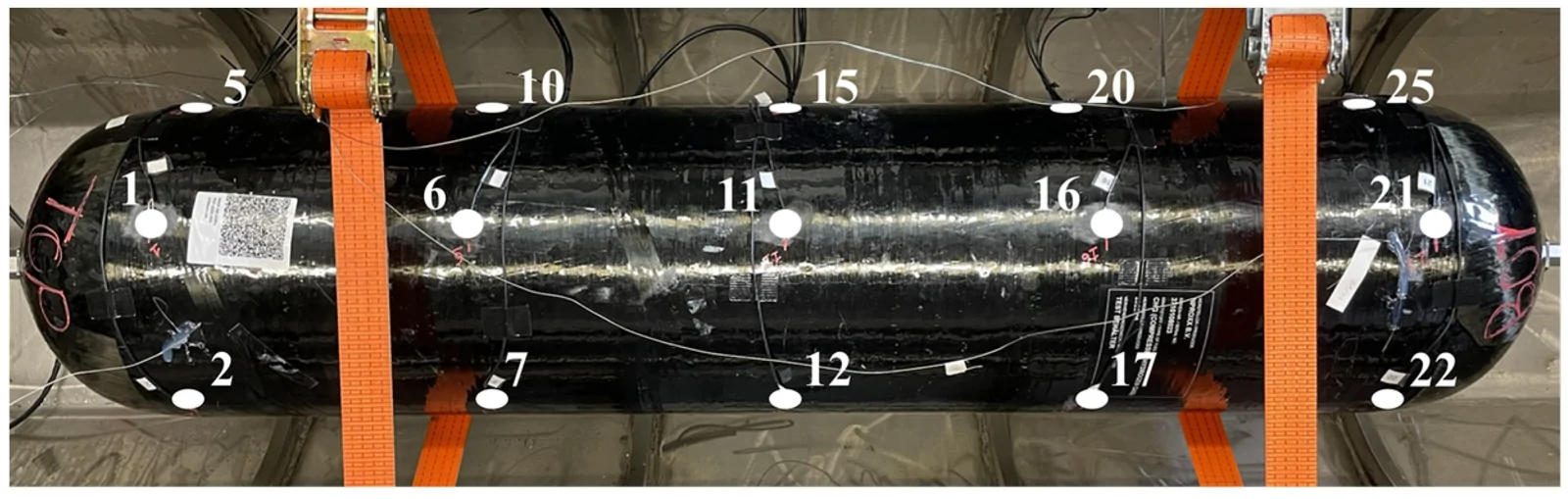
Top view of the COPV inside the high-pressure test facility, showing the installed sensors and their corresponding sensor numbers.

The investigated specimen is a COPV with a length of 1670 mm and a diameter of 352 mm, designed for a nominal operating pressure of 700 bar and manufactured by NPROXX GmbH (Alsdorf, Germany). According to the manufacturer, the Type IV pressure vessels employ a carbon-fiber-reinforced composite structure manufactured by wet filament winding. The vessel was equipped with 25 piezoelectric DuraAct patch sensors (P-876K025, PI Ceramics, Lederhose, Germany). The whole set-up was positioned within a burst chamber (Figure 1).

**Figure 2:**
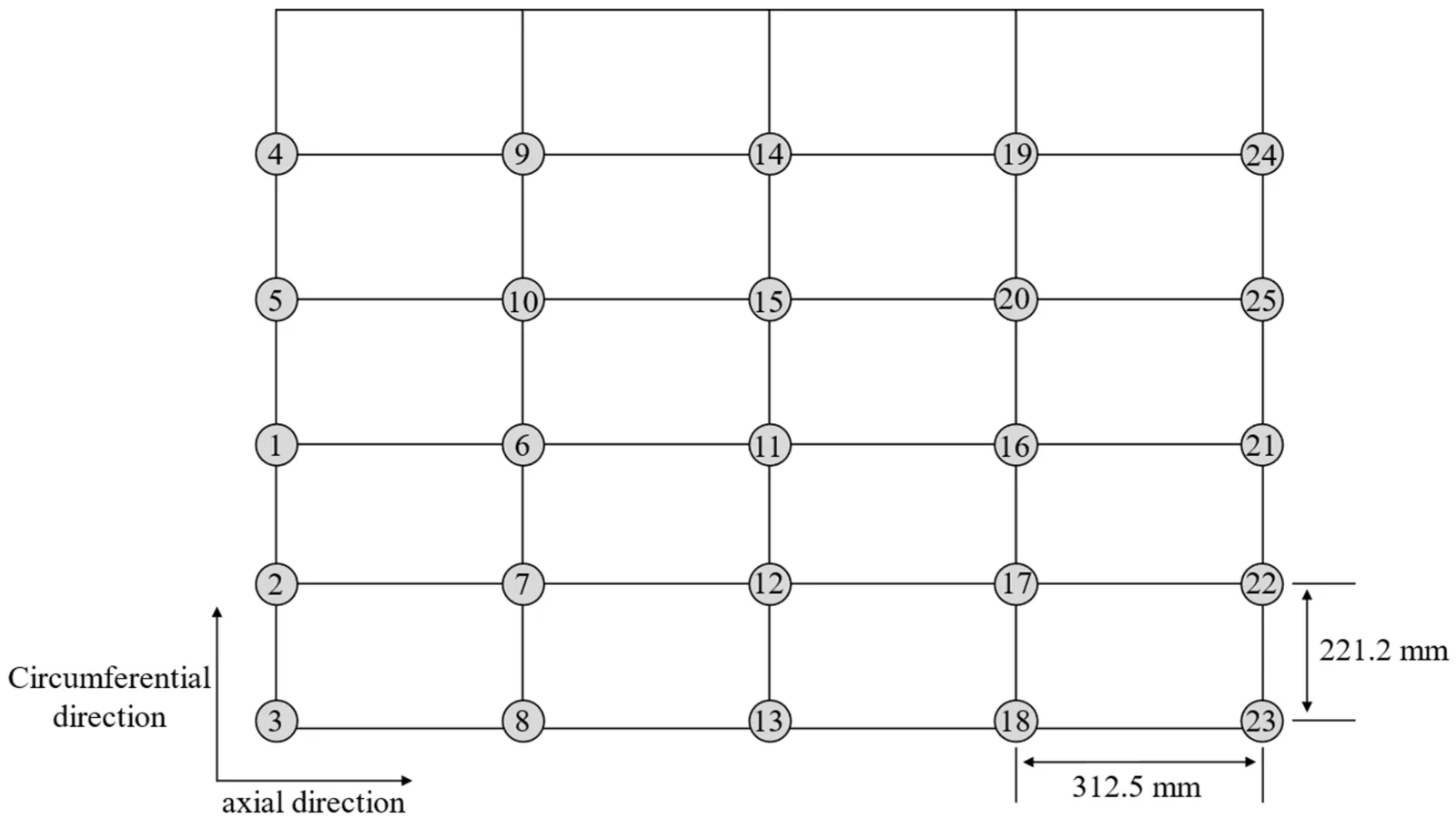
Overview of the sensor network on the unwrapped cylindrical surface of the COPV without dome areas. Sensors, numbered 1–25, are arranged in five circumferential rings.

The sensors were distributed across five circumferential rings, featuring an equal circumferential spacing of 221.2 mm and an axial spacing of 312.5 mm (Figure 2). Attachment to the vessel surface was achieved using an epoxy-based adhesive. Operating conditions were continuously monitored using DAkkS-calibrated instrumentation (P3 Top Class (P3TC), HBM and HSRTD-3-100-A-10M, OMEGA Engineering Inc., Norwalk, CT, USA).

Ultrasonic excitation and signal acquisition were carried out with a Vantage 64 LF system by the manufacturer Verasonic (Kirkland, WA, USA). Measurements were obtained in a pitch–catch configuration using discrete excitation frequencies of 60, 120, 180, 260, and 300 kHz, as well as a broadband chirp signal covering the range from 60 to 300 kHz.

**Figure 3:**
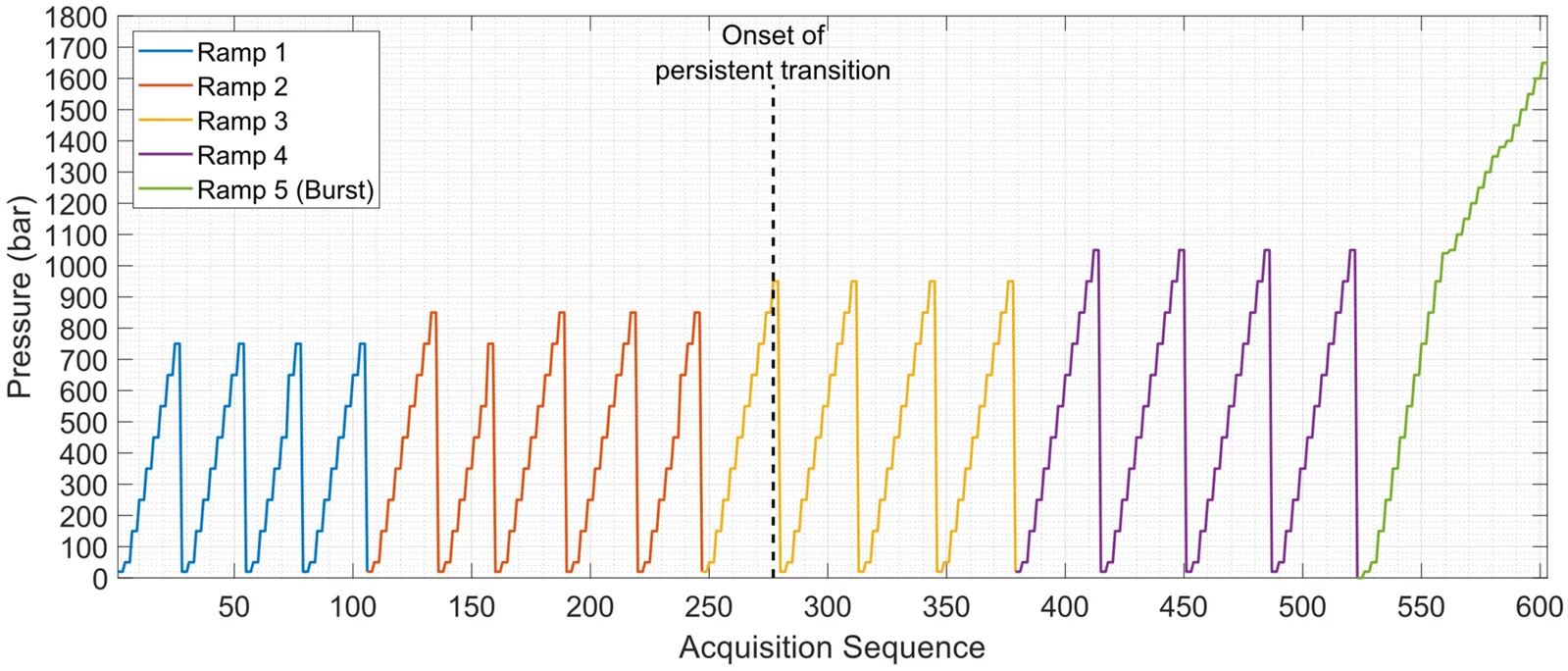
Pressure loading protocol used during the experimental campaign. Ramps 1–4 consist of repeated pressure cycles with increasing maximum pressure levels, while Ramp 5 represents the final burst experiment. As the second pressure cycle of Ramp 2 was not successfully acquired, an additional pressure cycle was included in this ramp. UGW measurements were acquired at every pressure level for all investigated excitation frequencies. The dashed line marks the first measurement at which the persistent structural transition was detected.

Four stepwise pressurization tests were carried out. The maximum pressures were 750 bar (Ramp 1), 850 bar (Ramp 2), 950 bar (Ramp 3), and 1050 bar (Ramp 4). The manufacturer specifies a safety limit of 1050 bar. Ramps 1–3 remained below this limit, while Ramp 4 reached the safety limit. The pressurization tests were followed by the final burst experiment (Ramp 5). Except for the burst experiment, each ramp was repeated over several pressure cycles, as shown in Figure 3. Starting from an initial pressure of 20 bar, the pressure was first increased to 50 bar and subsequently raised by increments of 100 bar at a constant rate of 50 bar/min. After each increment, a holding phase of six minutes at constant pressure was applied. During this period, after a stabilization time of three minutes, three repeated UGW measurements were performed for each combination of pressure level and excitation frequency, as well as for the chirp excitation. A single measurement consists of acquiring UGW signals for all propagation paths within the sensor network. For the 25-transducer sensor network used in this work, this corresponds to 600 pitch-catch signals (25 transmitting transducers × 24 receiving transducers). The vertical dashed line in Figure 3 marks the first measurement at which the persistent transition was detected, as identified and discussed in Section 4.1. All measurements acquired after the vessel pressure first reached 950 bar show a persistent change in the structural response and hence belong to the post-transition state.

After completion of the repeated pressurization ramps, a final burst experiment (Ramp 5) was performed. Unlike Ramps 1–4, which were repeated over several pressure cycles, Ramp 5 consisted of a single pressure cycle. The vessel was pressurized following the same loading procedure up to 1050 bar, except that Ramp 5 started from the initial pressure level of zero bar rather than continuing from the previous ramp. Beyond the manufacturer-specified safety limit of 1050 bar, the loading was continued with additional pressure levels, as shown in Figure 3, until burst failure occurred at 1713 bar. UGW measurements were acquired at each constant pressure level using the same measurement protocol.

## 3. Materials and Methods

Signal processing starts with a Butterworth bandpass filter with ±15 kHz bandwidth centered at the excitation frequency [24]. The filtering suppresses out-of-band noise while the fundamental A0 and S0 Lamb wave modes remain isolated [25]. This ensures that only the energy associated with the excitation frequency is assessed, which minimizes unwanted variance in the baseline data. The next step consists of isolating the signal’s first arrival to exclude boundary reflections and late-arrival interference [26] by applying a temporal window from the signal’s onset to 480 μs. During the initial development of the ML pipeline, different feature extraction methods were explored. Four feature extractors were then investigated: Best Daubechies Wavelet (BDW), Best Fourier Coefficients (BFC), Adaptive Linear Approximation (ALA), and Statistical Moments, as described in [27]. They were selected because they describe UGW signals in different approaches. BDW analyzes localized signal characteristics in the time-frequency domain. BFC describes the signal in the frequency domain. ALA and Statistical Moments operate directly on the time-domain waveform. Following that, the extracted features are then standardized to mitigate scaling issues and enable their comparability across subsequent ML stages [28].

We first compared the standardized features by projecting them into a Principal Component Analysis (PCA) space and visualizing up to the first three Principal Components (PCs) [29], as those components highlight the dominant variations in the extracted features [30]. A clear separation of the resulting clusters indicates that the extracted features capture changes associated with the structural state of the COPV. The PCA visualization helped identify measurements belonging to the post-transition structural state resulting from overpressurization and to separate them from measurements of the pre-transition state.

The kNN algorithm is trained only on the pre-transition dataset to establish a semi-unsupervised novelty detector [31]. The trained model is subsequently applied to a test set consisting of the retained pre-transition measurements and post-transition measurements. Each measurement is first characterized by identifying its k nearest neighbors using the Euclidean distance metric [20]. The novelty score is then calculated as the sum of the Euclidean distances to these k nearest neighbors [29].

To separate baseline states and post-transition states, an adaptive threshold is calculated following Equation (1) with a sensitivity factor x, using the mean μ and standard deviation σ of the healthy baseline scores [20].

(1) Threshold=μ+(x·σ),

The final step is to validate the model using the LOGO-CV approach [32]. In this method, each group represents a specific pressure ramp. For each validation fold, one pre-transition ramp is retained for testing, and the remaining pre-transition groups are used for model training. To evaluate the detector on both pre-transition and post-transition conditions, a post-transition ramp is evaluated together with the retained pre-transition test ramp during the evaluation phase. Table 1 illustrates the different permutations used for these validation folds. For each validation fold, standardization and all model-related fitting steps, including threshold estimation, were performed exclusively using the corresponding training data.

**Figure 4:**
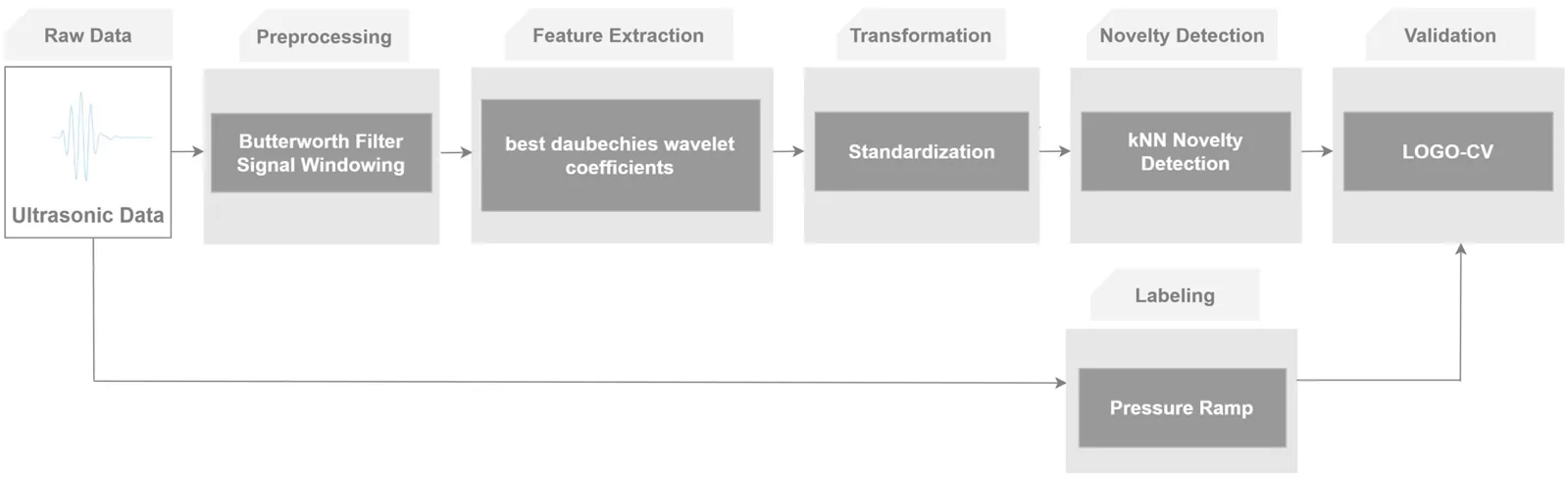
Workflow of the semi-unsupervised ML pipeline, illustrating the sequence from raw UGW acquisition and exemplary BDW feature extraction to kNN-based novelty detection and LOGO-CV validation.

The overview of the proposed ML pipeline is illustrated in Figure 4. The pipeline begins with preprocessing the acquired UGW data. This step is followed by feature extraction, and finally, novelty detection is validated using the LOGO-CV approach.

The LMT-ML-Toolbox [27] is employed to automatically evaluate the performance of the investigated feature extractors. In this process, hyperparameters of the kNN novelty detector are initially set to values that have turned out to be suitable for an initial inspection [20,33]. Hence, the k-Number is set to 3, the standard deviation factor is set to 3.36, while the Euclidean distance is applied [20]. Once the best-performing feature extractor is selected, hyperparameter optimization is then performed to identify the set-up yielding the highest performance.

## 4. Results

### 4.1. Feature Extraction Visualization Using PCA

**Figure 5:**
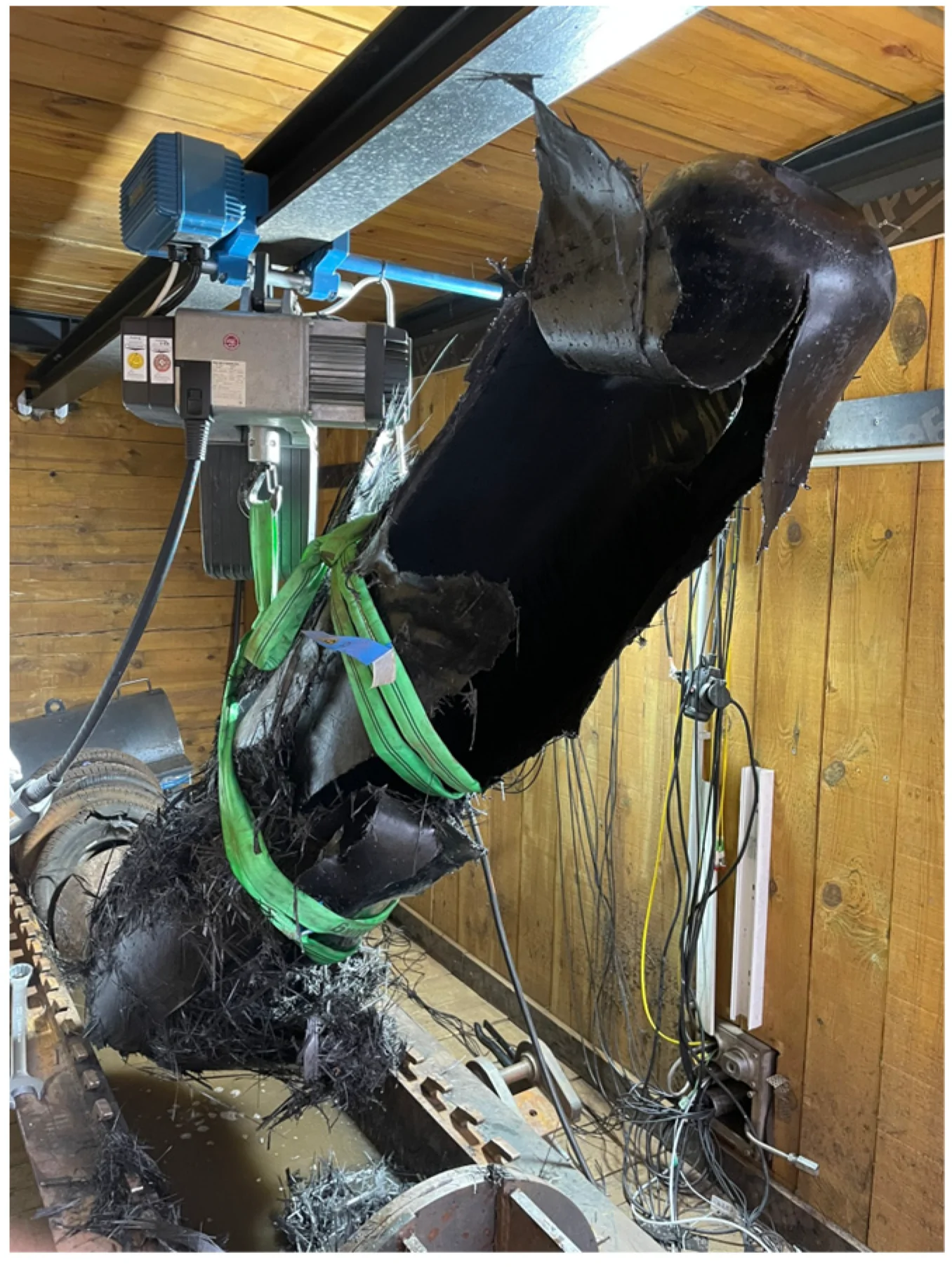
COPV after burst failure. The vessel shown corresponds to the final burst experiment (Ramp 5), in which failure occurred at a pressure of 1713 bar. The photograph suggests that the rupture occurred within the cylindrical section of the vessel.

**Figure 6:**
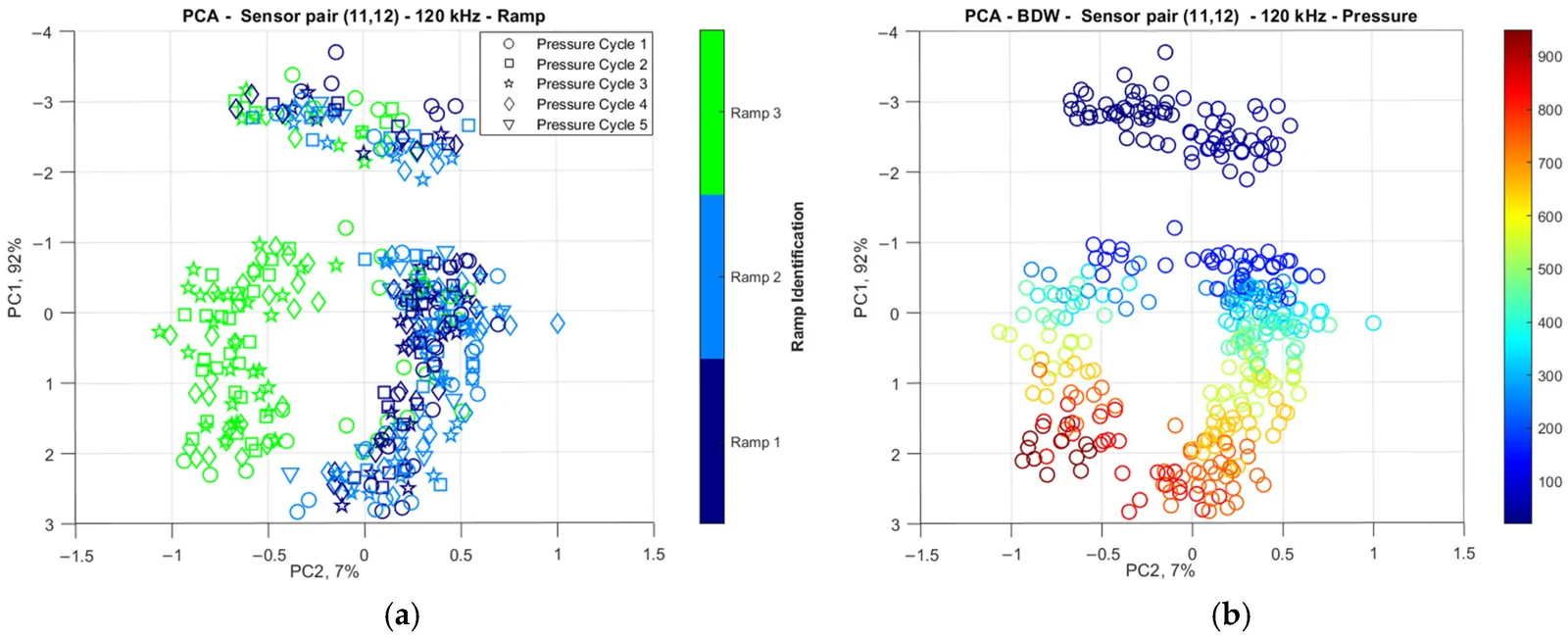
PCA projection of the BDW feature space for sensor pair 11–12 at an excitation frequency of 120 kHz, including measurements from Ramps 1–3. (**a**) Measurements colored by pressure ramp. (**b**) The same projection colored by applied pressure. The paired visualization is used to investigate the feature evolution prior to exceeding the manufacturer’s specified pressure limit.

Before defining the novelty classes, the extracted feature spaces were standardized and inspected using PCA. The feature extractor comparison is illustrated for sensor pair 11–12. This pair was selected a priori because its propagation path is located in the cylindrical mid-section of the pressure vessel, where high hoop stresses are expected under internal pressure [34]. The selection was therefore based on the expected mechanical response of this vessel region and not on the subsequently observed classification performance or burst location. The burst eventually occurred in the same cylindrical region (Figure 5). It therefore provides a suitable example for illustrating the structural changes captured by the investigated feature extractors. Several feature extractors were evaluated, including ALA, statistical moments, BFC, and BDW. Their corresponding PCA projections are provided in Appendix A.1, Appendix A.2 and Appendix A.3. Among the investigated methods, BDW produced the clearest separation between the response regions while preserving a compact representation of the measurements (Figure 6). The subsequent analysis therefore focuses on the BDW features.

The interpretation begins with the initial assumption that Ramps 1–3 belong to the pre-transition state. This assumption follows from the experimental procedure, since the manufacturer specifies 1050 bar as the upper pressure limit before the final overload sequence.

Figure 6 shows the PCA projection of Ramps 1–3. Both PCAs display the same feature space. In Figure 6a, the measurements are colored according to the pressure ramp, whereas Figure 6b uses the applied pressure. Most measurements occupy a common response region. However, the ramp-colored representation reveals that several measurements acquired in the latter part of Ramp 3 are distinct from the remaining data. The pressure-colored representation shows that these measurements correspond to pressures above 850 bar. The first separated measurement is recorded at 950 bar. At this stage, the observation is unexpected because Ramp 3 does not exceed the manufacturer-defined pressure limit. The transition was identified by an unsupervised PCA of the extracted features. In this step, we did not employ predefined class labels. The coloring is added only to visually interpret the clustering and is not used in the transition identification or model development. The resulting transition definition is subsequently used as the operational separation between pre-transition and post-transition measurements for the quantitative evaluation. An excitation frequency of 120 kHz was selected for the PCA and the subsequent model development. The choice reflects the trade-off between improved sensitivity to smaller structural changes at higher frequencies and better separation of the A0 and S0 modes at lower frequencies. A comparatively low excitation frequency was therefore preferred for the detailed analysis. Therefore, the selection was made independently of the subsequent novelty detection results [15,24].

**Figure 7:**
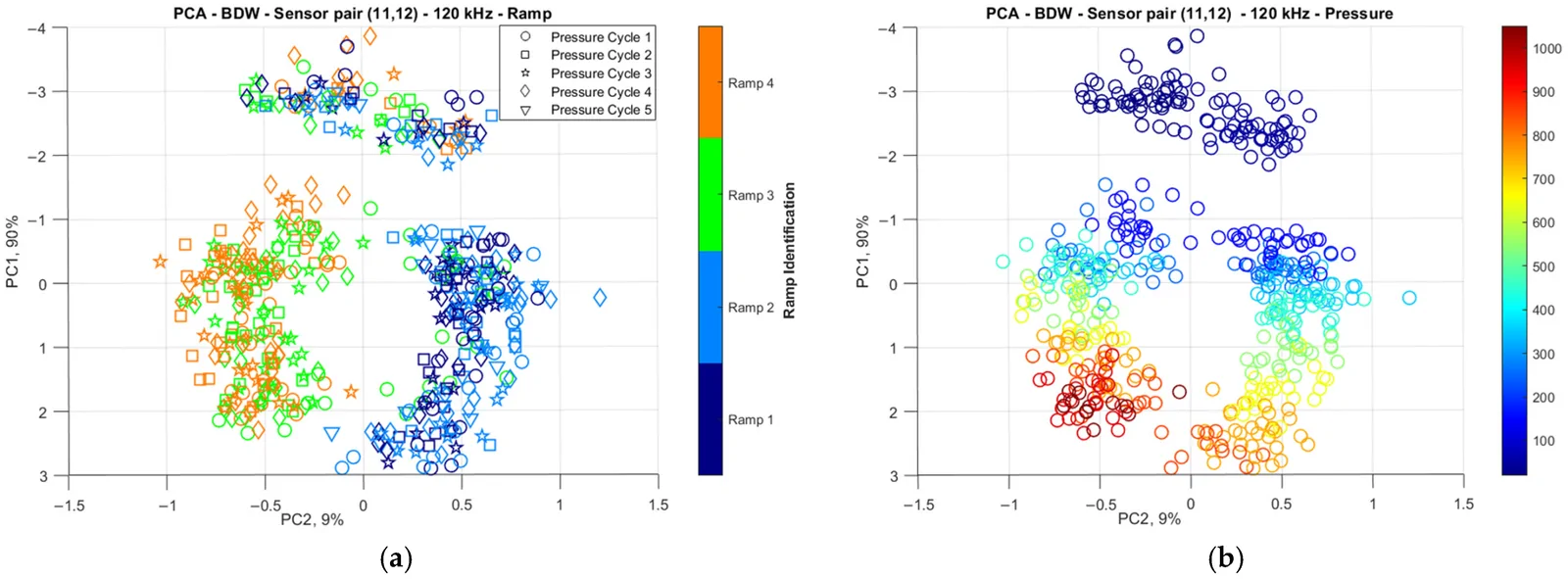
PCA projection of the BDW feature space for sensor pair 11–12 at an excitation frequency of 120 kHz, including measurements from Ramps 1–4. (**a**) Measurements colored by pressure ramp. (**b**) The same PCA projection colored by applied pressure. The additional loading cycle enables a comparison of the separated measurements from Ramp 3 with the response observed after loading beyond the manufacturer’s specified pressure limit.

To better understand this behavior, Ramp 4 is added to the PCA (Figure 7). Again, the same projection is shown using the two coloring schemes. The measurements that separated during Ramp 3 now coincide with the response region occupied by Ramp 4. This suggests that the change observed in Ramp 3 is related to the structural evolution that becomes more pronounced during the subsequent loading cycle. At the same time, the pressure-colored projection shows that the separation cannot be attributed solely to the instantaneous pressure since the measurements remain in the new response region, even at pressures below 950 bar.

**Figure 8:**
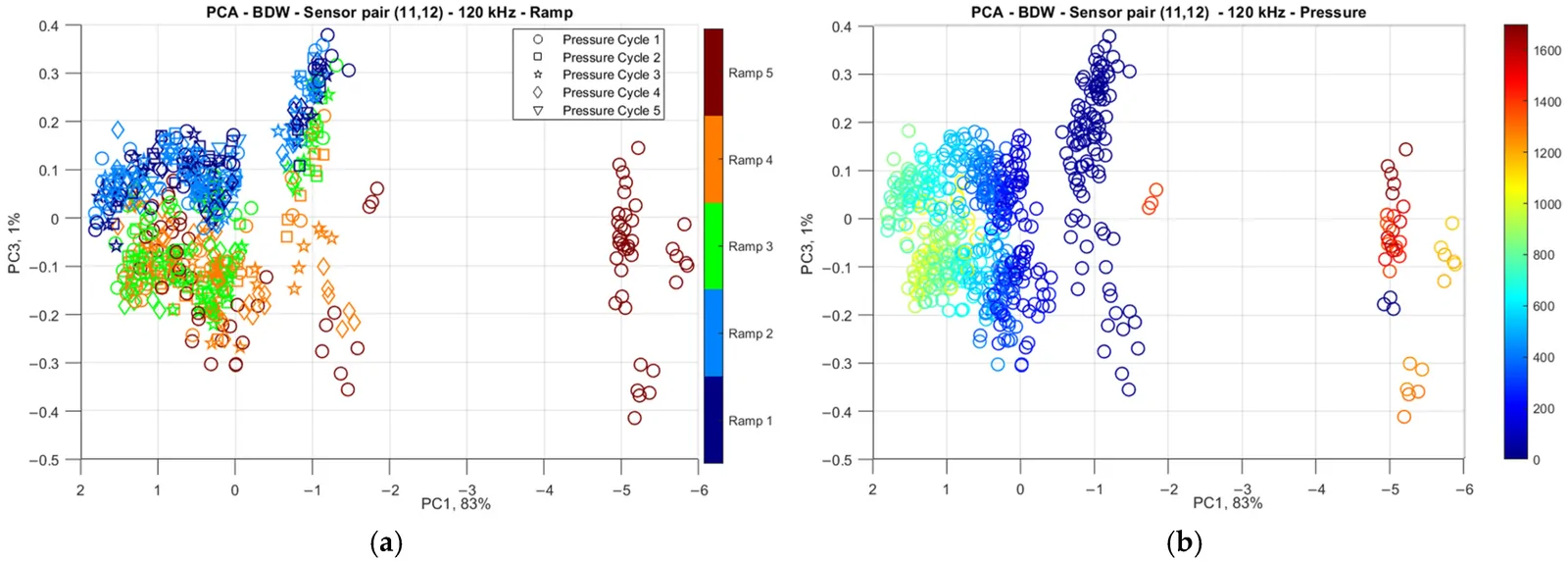
PCA projection of the BDW feature space for sensor pair 11–12 at an excitation frequency of 120 kHz, including all pressure ramps and the final burst experiment. (**a**) Measurements colored by pressure ramp. (**b**) The same projection colored by applied pressure. The complete dataset illustrates the evolution of the feature space throughout the entire pressure experiment.

The complete experiment is finally shown in Figure 8, including the burst experiment (Ramp 5). The measurements from the later part of Ramp 3 continue to cluster with those from Ramps 4 and 5, whereas the earlier measurements from Ramps 1–3 remain within the initial response region. The PCA therefore indicates a persistent transition in the UGW response rather than a temporary pressure-dependent effect. Based on this observation, the measurements up to 850 bar are assigned to the pre-transition state, while the first separated measurement at 950 bar and all subsequent measurements are assigned to the post-transition state.

### 4.2. Model Configuration

Several design decisions preceded the final model configuration. The feature extraction method was evaluated first because it defines the information available to the novelty detector. The selected feature representation was subsequently used to configure the kNN detector. Finally, the decision threshold was optimized for the resulting model.

#### 4.2.1. Feature Extractor Selection

To assess the contribution of the feature representation independently of the novelty detector, all investigated feature extractors were evaluated using the same kNN configuration. The PCA presented in the previous section provides an initial qualitative assessment of the extracted feature spaces. To quantitatively compare the investigated feature extraction methods, the novelty detection framework described in Section 3 was applied to the features generated by each extractor for sensor pair 11–12.

The extracted features were again standardized and evaluated using the kNN novelty detector described in Section 3. Throughout the feature extractor comparison, the detector configuration was kept identical for all investigated methods. Euclidean distance, three nearest neighbors, and the corresponding decision threshold described in Section 3 were used for all evaluations. Consequently, only the parameters controlling the individual feature representations were varied.

For each feature extractor, the parameter controlling the feature representation was varied while all remaining model parameters were kept unchanged. For BDW, the number of retained wavelet coefficients was investigated. For ALA, different segment lengths were evaluated. For BFC and Statistical Moments, the number of extracted features was varied accordingly. The investigated parameter ranges were selected based on preliminary experiments to cover representative feature complexities while avoiding unnecessarily small or excessively large feature sets.

The performance of each configuration was evaluated using LOGO-CV. As the dataset consists of substantially more post-transition than pre-transition measurements, balanced accuracy (BA) was used as the optimization criterion instead of overall accuracy. BA is defined as:

(2) $$BA=\frac{TPR+TNR}{2}$$

In this study, true positive rate (TPR) represents the model’s ability to correctly identify measurements belonging to the post-transition structural state, whereas true negative rate (TNR) quantifies the correct identification of measurements acquired in the normal (pre-transition) structural state. This performance measure was adopted because the TNR describes the model’s ability to avoid false alarms within the pre-transition range, whereas the TPR describes its ability to detect post-transition states. In this application, avoiding false alarms is considered more critical. On the other hand, once a sufficient number of post-transition measurements have been correctly identified, the transition is then considered detected. The balanced accuracy was determined for each LOGO-CV fold and subsequently averaged across all folds. The standard deviation was additionally reported to evaluate the consistency of the model between the individual validation groups.

Table 2 summarizes the best-performing parameter configuration of each feature extractor. The complete optimization results for all investigated parameter configurations are provided in Appendix B.1. Among the evaluated methods, BDW achieved the highest mean balanced accuracy of 96.12% with a standard deviation of 2.23%, confirming the separation already suggested by the PCA visualization. ALA yielded a comparable balanced accuracy of 95.13% but exhibited greater variability across the validation folds. In contrast, BFC and Statistical Moments yielded considerably lower balanced accuracies and substantially higher standard deviations, indicating reduced robustness of the extracted feature representations.

For the BDW extractor, retaining five wavelet coefficients provided the highest balanced accuracy. Although configurations with a larger number of coefficients produced a slightly lower standard deviation, they consistently reduced the mean balanced accuracy. BDW, with five retained wavelet coefficients, was therefore selected for subsequent hyperparameter optimization.

#### 4.2.2. kNN Hyperparameter Optimization

Following the selection of the BDW feature extractor, the hyperparameters of the kNN novelty detector were optimized. The first investigated parameter was the distance metric, as it defines how the similarity between feature vectors is quantified and therefore directly influences the neighborhood construction used for novelty detection. All remaining detector parameters were kept unchanged during this evaluation. The performance of each distance metric was assessed using the LOGO-CV procedure described in Section 3. To obtain a representative assessment of the model performance for the investigated kNN hyperparameters, the evaluation was performed for three sensor pairs distributed over different COPV regions. While sensor pair 11–12 is located close to the cylindrical mid-section, sensor pair 6–10 is located between the cylindrical section and the vessel dome, and sensor pair 1–2 is located close to the dome. The additional sensor pairs were selected to investigate the influence of sensor position and the expected variation in sensitivity across these vessel regions. The mean BA across all sensor pairs and validation folds was used as the primary selection criterion, while the corresponding standard deviation was considered to assess the robustness of the individual distance metrics. The obtained results are summarized in Table 3. Euclidean and Chebyshev distance yield the highest balanced accuracies. Since both metrics showed nearly identical performance, they were both considered in the subsequent optimization of the number of nearest neighbors.

After selecting the distance metric, the effect of the number of nearest neighbors on the novelty detection performance was investigated. The detector was evaluated for the selected 10 values of k between 1 and 35 while keeping all other parameters unchanged. Performance was assessed using the LOGO-CV procedure described in Section 3. Mean balanced accuracy was used to compare the different configurations, and its standard deviation was considered as an indicator of robustness. The corresponding results are listed in Table 4.

Table 4 and Table 5 present the balanced accuracy and true negative rate obtained for different values of k using the Euclidean and Chebyshev distance metrics. The true negative rate increased with increasing k  for both distance metrics, whereas the balanced accuracy gradually decreased. Since the detector is intended to identify persistent structural transitions, false alarms should be avoided whenever possible. The true negative rate was therefore considered more important than the balanced accuracy during the parameter selection. The Chebyshev distance metric with k=10 was selected because it achieved a mean true negative rate of 93.02% while still providing a balanced accuracy of 77.47%. This was also the first configuration for which sensor pair 11–12 reached a true negative rate of 100%. Sensor pair 11–12 is located in the cylindrical mid-section, which is strongly affected by the pressure-induced stress state and therefore represents one of the most relevant propagation paths for monitoring structural changes. Larger values of k increased the true negative rate only slightly, whereas the balanced accuracy continued to decrease.

#### 4.2.3. Sensitivity Factor Optimization

The sensitivity factor x was varied between 0 and 8.5, while the previously selected Chebyshev distance metric and k=10 were kept unchanged. The corresponding results are summarized in Table 6. Increasing the sensitivity factor raised the novelty threshold and therefore reduced the number of pre-transition measurements that were incorrectly classified as novel. Consequently, the mean true negative rate increased with increasing x. At the same time, the balanced accuracy gradually decreased because the higher threshold also reduced the detector’s sensitivity to post-transition measurements.

The highest mean balanced accuracy of 81.57% was obtained for a sensitivity factor of x=1.0. However, the corresponding mean true negative rate was only 78.54%, indicating that a considerable number of pre-transition measurements were still classified as novel. Since the primary objective of the novelty detector is to avoid false alarms during normal operation while reliably identifying the persistent structural transition, a high true negative rate was considered more important than maximizing the balanced accuracy alone.

A sensitivity factor of x = 3.25 was selected during hyperparameter optimization after evaluating the LOGO-CV results, using the trade-off between true negative rate and balanced accuracy. At this value, the mean true negative rate reached 92.70%, with a standard deviation of 15.84%, while the mean balanced accuracy remained at 77.70%. More importantly, this was the first investigated operating point for which the representative sensor pair 11–12 achieved a true negative rate of 100%, indicating that no false alarms occurred for the sensor pair located closest to the pressure-induced structural transition. Although higher sensitivity factors further increased the mean true negative rate, these improvements were comparatively small and were accompanied by a continuous reduction in balanced accuracy. The selected operating point therefore provides a suitable compromise between minimizing false alarms and maintaining sufficient sensitivity to reliably detect the persistent structural transition.

### 4.3. Visualization of the Novelty Detection Results

The quantitative evaluation presented in the previous section summarizes the classification performance of the novelty detector. However, these metrics do not illustrate how the novelty response evolves during the pressure ramps or how the detected transition relates to the applied pressure. The novelty scores are therefore visualized together with the corresponding internal pressure in the order in which the measurements were acquired. This representation allows the detector response to be directly related to the pressure history and demonstrates whether elevated novelty scores persist after the pressure is subsequently reduced.

**Figure 9:**
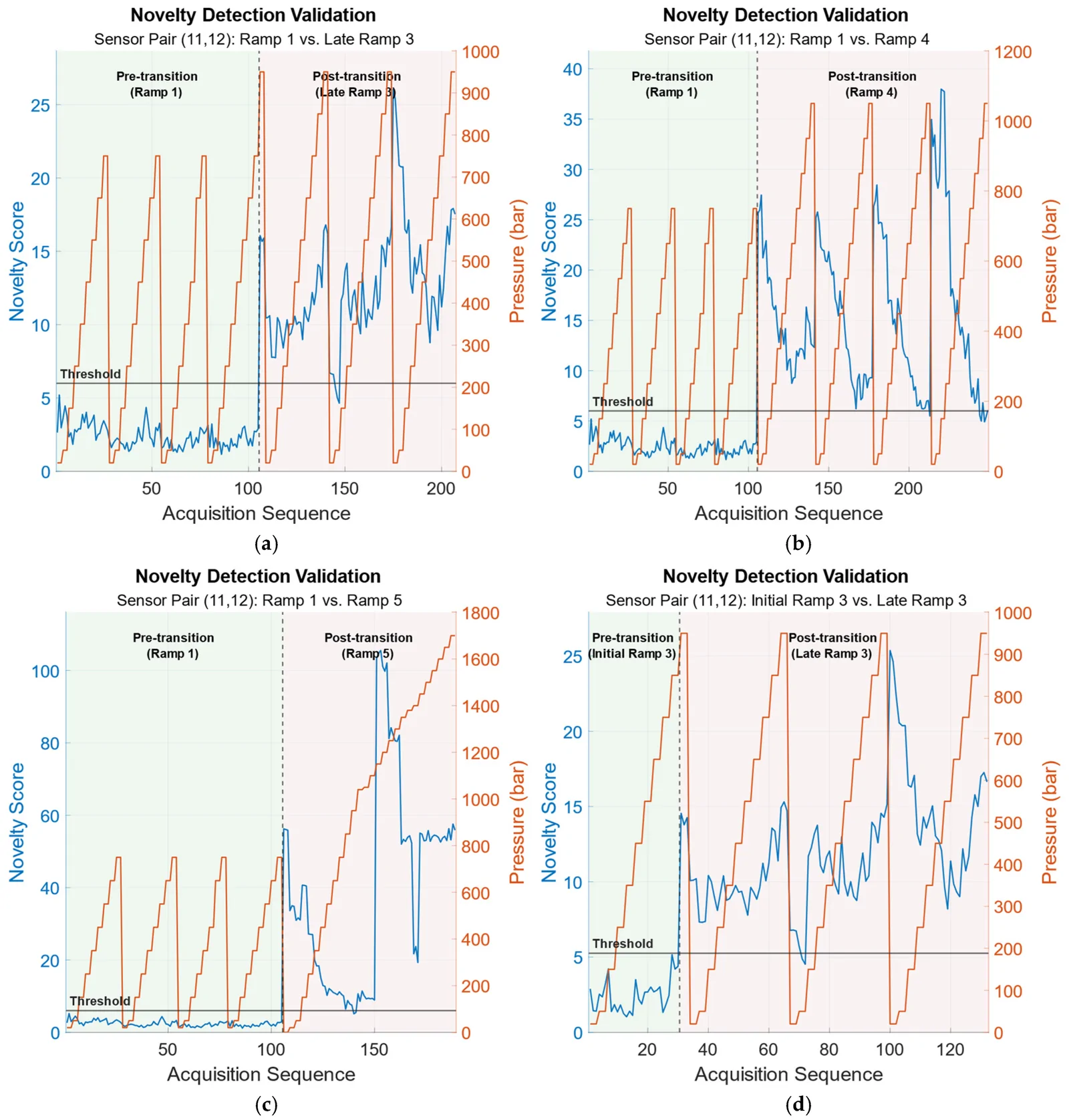
Novelty detection results for sensor pair 11–12 using different validation scenarios as specified in Table 1: (**a**) Ramp 1 vs. the post-transition measurements of Ramp 3, corresponding to Fold 1; (**b**) Ramp 1 vs. Ramp 4, corresponding to Fold 2; (**c**) Ramp 1 vs. Ramp 5 (burst), corresponding to Fold 3; and (**d**) the initial measurements of Ramp 3 vs. the post-transition measurements of Ramp 3, corresponding to Fold 7. The blue curve represents the novelty score, and the orange curve represents the applied pressure. The vertical dashed line marks the beginning of the post-transition measurements.

The following discussion focuses on sensor pair 11–12 because it crosses the cylindrical mid-section of the vessel, where the final burst occurred, and exhibited the highest classification performance. Figure 9 presents representative LOGO-CV validation folds. Figure 9a–c evaluate Ramp 1 as an independent pre-transition test group together with the post-transition measurements from Ramps 3, 4, and 5, respectively. For these validation folds, the novelty detector was trained exclusively on the remaining pre-transition measurements consisting of Ramp 2 and the initial section of Ramp 3. Figure 9d focuses on the transition within Ramp 3 itself. In this case, the detector was trained on the pre-transition measurements consisting of Ramps 1 and 2 and subsequently evaluated on the initial and late sections of Ramp 3.

The results shown in Figure 9a–c demonstrate a consistent separation between the pre-transition and post-transition measurements. The novelty scores obtained for Ramp 1 remain predominantly below the calculated threshold, whereas the scores of the post-transition measurements exceed the threshold over large parts of the acquisition sequence. Similar behavior is observed for all three post-transition ramps, indicating that the detector does not merely recognize a specific pressure cycle but consistently identifies the persistent structural transition across independently acquired experiments.

Figure 9d provides additional insight into the onset of the transition. Since both test subsets originate from the same pressure ramp, differences caused by separate experimental runs are eliminated. The novelty scores remain below the threshold throughout the initial section of Ramp 3 but increase after the vessel has been exposed beyond 850 bar. The transition first becomes evident at the 950 bar measurement level and remains detectable during the subsequent pressure cycles, even when the internal pressure is reduced before increasing again. This observation indicates that the detector responds to a persistent change in the UGW response rather than to the instantaneous pressure alone. The behavior is consistent with the PCA visualization discussed previously and further supports the existence of an irreversible structural transition.

The remaining LOGO-CV validation folds show comparable behavior and are therefore provided in Appendix B.2, Appendix B.3 and Appendix B.4. Together, these results demonstrate that the proposed novelty detector reliably distinguishes pre-transition from post-transition measurements while maintaining consistent performance across independent pressure ramps and validation folds.

## 5. Discussion

The experimental results indicate a persistent structural transition after the vessel had been exposed beyond 850 bar. On the one hand, this observation is supported by the PCA clusters shown in Figure 7, where the measurements form a distinct post-transition cluster. On the other hand, the same observation is confirmed by the novelty detector, whose novelty scores remain above the decision threshold even after the vessel pressure has returned to the normal operating level. Together, these observations show that the employed BDW feature extractor captures persistent structural changes rather than only the reversible elastic response caused by the applied pressure.

Following the identification of the transition, its relation to the applied pressure was examined, where the first separation was observed at the 950 bar measurement level. Pressure information was not included in either analysis. Both the PCA and the novelty detector were based exclusively on the measured UGW signals, while the pressure values were considered afterwards to relate the detected transition to the loading history. The transition at 950 bar was therefore identified from changes in the measured structural response rather than from a predefined pressure threshold. Its physical origin, however, cannot be determined from the present measurements, since no additional inspection was performed at this stage of the experiment. In this study, the term irreversible structural transition refers to a persistent change in the measured UGW response that remains after unloading and does not imply the identification of a specific physical damage mechanism. Nevertheless, the persistence of the changed UGW response after unloading indicates that the transition cannot be explained solely by the reversible elastic response to pressure. A persistent structural change can modify the effective stiffness and local mechanical properties of the composite [35]. Such changes can in turn affect guided-wave propagation, including velocity, attenuation, scattering, and mode content, thereby altering the time-frequency characteristics represented by the BDW coefficients [11,13]. The present measurements, however, do not allow the underlying physical mechanism to be identified. The quantitative performance reported in this study is therefore conditional on the data-driven transition definition obtained from the UGW measurements and should not be interpreted as validation against an independent physical damage ground truth. The results show that a persistent change in the structural response became detectable before the manufacturer-specified safety limit of 1050 bar was reached. The clustering of measurements acquired at the manufacturer-specified safety limit together with measurements obtained above this limit further supports the interpretation that the observed transition reflects a persistent structural alteration rather than a purely pressure-dependent effect.

Not all sensor pairs responded equally to the structural transition. Sensor pair 11–12 produced the highest balanced accuracy and showed the clearest separation during Ramp 3. In contrast, sensor pair 1–2 was less sensitive to the onset of the transition but still distinguished the post-transition state from the baseline. This behavior agrees well with the expected stress distribution in Type IV COPVs, where the cylindrical section experiences the highest hoop stresses under internal pressure [36]. These observations highlight that sensor placement has a strong influence on detection performance.

We evaluated the novelty detector for each sensor pair separately. Sensor pair 11–12 consistently produced the best classification performance and detected the transition in every pressure ramp. This raised another question. The sensor network had originally been designed for damage localization, where many propagation paths are required. For transition detection alone, however, the results suggest that this may not be necessary. At least for the investigated vessel type, one carefully selected sensor pair already provided reliable detection of the persistent transition. A reduced sensor configuration would simplify the measurement system and reduce the required instrumentation. The full sensor network is still needed when the objective is to determine where the structural change occurred rather than only whether it occurred.

An interesting observation was made when the individual pressure ramps were excluded from the training data. Omitting Ramp 1 resulted in the largest decrease in detection performance for the less sensitive sensor pairs 6–10 and 1–2, whereas the best-performing sensor pair (11–12) remained comparatively robust (Figure 9 and Appendix B.2, Appendix B.3 and Appendix B.4). Excluding the later pressure ramps had a smaller effect. Ramp 1 therefore appears to play an important role in defining the reference state of the novelty detector.

Most existing machine learning approaches for UGW-based damage assessment rely on supervised classification or regression and therefore require measurements representing the investigated damage conditions during model training. A direct quantitative comparison with these approaches is difficult because the investigated structures, damage conditions, sensor configurations, and validation procedures differ. More importantly, the present study addresses a different learning problem, since the novelty detector is trained exclusively on measurements representing the healthy structural condition and does not require examples of the subsequent structural transition [15,24].

A further advantage of the proposed method is that it only requires measurements from the baseline state. Collecting representative data after structural degradation is often impractical because deliberately damaging safety-critical structures is difficult and costly [37]. Reference measurements in the normal operating range, in contrast, are usually available during commissioning. Under these conditions, an unsupervised novelty detector offers a practical alternative to supervised classification approaches. The consistently high true negative rates obtained in this work are particularly relevant for hydrogen storage systems, where false alarms may lead to unnecessary inspections and operational downtime.

The present validation is limited to independently conducted pressure ramps of a single COPV specimen. The LOGO-CV therefore evaluates generalization across loading sequences, but not across different vessels. The current results should consequently be regarded as a proof-of-concept. Validation using additional COPVs with different manufacturing and loading histories is required before broader generalization to hydrogen storage vessels can be established.

The present experiments generated the structural changes by controlled overpressurization. This might suggest that pressure monitoring alone would be sufficient. A pressure sensor can indeed indicate when the nominal operating pressure of 700 bar or the manufacturer-specified safety limit of 1050 bar has been exceeded. However, pressure and structural condition are not the same quantity. Pressure describes the applied load, whereas the proposed method evaluates the structural response. Consequently, the detector still identifies the post-transition state after the pressure has returned to the normal operating range. More importantly, overpressurization is only one possible source of persistent structural changes. Similar modifications may result from repeated pressure cycling, impact events, manufacturing imperfections, environmental influences, hydrogen exposure, or long-term material aging. The proposed framework is therefore applicable beyond the specific loading scenario investigated in this study, provided that such mechanisms produce measurable changes in the UGW response.

The current framework detects whether the structural response has changed, but it does not quantify the extent of that change. Although larger novelty scores generally correspond to greater deviations from the healthy baseline, they cannot yet be interpreted as a measure of structural degradation. Developing such a relationship is an important topic for future work. A physically meaningful structural change metric could help distinguish between the first measurable transition observed by UGW and structural change that may affect the integrity of the vessel. Establishing this connection would move the method beyond binary novelty detection toward quantitative structural health assessment.

The present analysis was performed using a single excitation frequency and bandwidth. Future investigations should examine additional excitation frequencies and multi-frequency approaches to determine whether the sensitivity can be further improved. Environmental influences, including temperature variations [38] and gas-flow-induced noise during operations [39], should also be investigated. Finally, validating the approach under realistic operating conditions will be an important step toward continuous SHM of hydrogen storage systems.

## 6. Conclusions

This work shows that persistent structural changes in COPVs can be detected using an unsupervised novelty detection framework based on UGWs. The proposed methodology requires only measurements acquired from the healthy baseline state and reliably distinguishes them from measurements recorded after a structural transition induced by controlled overpressurization.

A persistent transition in the UGW response became detectable at the 950-bar measurement level, indicating that the underlying structural transition occurred after exposure beyond 850 bar. The transition was identified solely from the measured UGW signals, without incorporating pressure information into either the PCA or the novelty detection framework. The persistence of the detected transition after the vessel pressure had returned to the normal operating range further indicates a lasting structural change rather than a purely reversible pressure-induced elastic effect.

The experiments also showed that sensor placement strongly influences detection performance. Sensor pairs located near the eventual burst region achieved the highest detection accuracy, whereas lower sensitivity was observed in structurally reinforced regions. At the same time, reliable transition detection was already achieved using a single carefully selected sensor pair, indicating that the dense sensor network originally designed for localization is not necessarily required when the objective is limited to transition detection.

The presented framework provides a proof-of-concept for novelty-based SHM of hydrogen storage vessels because it does not require measurements from damaged structures for training. Although the current methodology reliably detects the occurrence of persistent structural changes, it does not yet quantify their severity. Establishing a physically meaningful relationship between novelty scores and structural degradation, together with validation under realistic operating conditions, will be an important step toward continuous SHM of COPVs.

## Figures and Tables

**Table 1:** Systematic overview of the LOGO-CV approach. The green cells denote the pre-transition pressure ramps used for training in each fold, while the blue cells indicate the corresponding unseen pre-transition and post-transition ramps utilized for model testing and novelty detection validation.

		Pre-Transition		
	Training Ramps	Ramp 2, 3	Ramp 1, 3	Ramp 1, 2
	Test Ramp	Ramp 1	Ramp 2	Ramp 3
**Post-** **Transition**	Ramp 3	Fold 1	Fold 4	Fold 7
	Ramp 4	Fold 2	Fold 5	Fold 8
	Ramp 5 (Burst)	Fold 3	Fold 6	Fold 9

**Table 2:** Performance comparison of the investigated feature extractors using their respective best-performing parameter configuration for sensor pair 11–12.

Extractor	Optimized Parameter	BA (%)	Std. Dev. (%)
BDW	5	96.12	2.23
ALA	30	95.13	3.21
BFC	20	86.79	9.76
Statistical Moments	10	65.73	10.02

**Table 3:** Balanced accuracy of the investigated kNN distance metrics for three representative sensor pairs. Mean BAs and the corresponding standard deviations are shown for all evaluated sensor pairs and LOGO-CV folds. The distance metrics are sorted in descending order according to their mean BA over all investigated sensor pairs.

Distance	BA for Sensor Pair 11–12 (%)	BA for Sensor Pair 6–10 (%)	BA for Sensor Pair 1–2 (%)	Mean BA of the Sensor Pairs (%)	Std. Dev. (%)
Euclidean	96.12	76.18	77.71	83.34	17.25
Chebyshev	97.37	76.57	75.93	83.29	17.97
Cityblock	76.87	64.74	67.18	69.60	11.83
Cosine	59.84	53.57	53.64	55.68	6.42
Mahalanobis	51.66	51.15	59.41	54.07	6.41
Spearman	52.19	55.60	53.80	53.86	5.79
Correlation	55.56	51.92	53.70	53.73	3.68
Hamming	50.00	50.00	50.00	50.00	0.00
Jaccard	50.00	50.00	50.00	50.00	0.00

**Table 4:** Performance of the Euclidean and Chebyshev distance metrics for different numbers of nearest neighbors (k).

	Euclidean Distance					Chebyshev Distance				
	BA (%) for Sensor Pairs			Mean BA (%)	Std. Dev. (%)	BA (%) for Sensor Pairs			Mean BA (%)	Std. Dev. (%)
k Values	11–12	6–10	1–2			11–12	6–10	1–2		
1	94.67	58.24	50.00	67.64	20.59	97.10	60.12	57.55	71.59	19.82
3	96.12	76.18	77.71	83.34	17.25	97.37	76.57	75.93	83.29	17.97
5	96.67	76.66	74.59	82.64	17.91	97.91	72.31	73.21	81.14	18.70
7	97.13	76.37	73.54	82.35	17.99	97.84	69.23	68.87	78.65	19.59
10	97.13	76.24	70.93	81.43	17.82	98.16	68.70	65.54	77.47	20.67
15	97.76	75.47	68.25	80.49	18.64	97.20	66.24	60.76	74.73	21.19
20	98.52	75.09	67.32	80.31	18.85	94.88	62.79	59.94	72.54	20.44
25	97.76	70.98	66.04	78.26	19.13	92.95	59.71	57.57	70.08	20.22
30	97.45	66.92	65.44	76.60	19.59	89.69	58.36	57.41	68.48	18.72
35	95.88	63.36	64.89	74.71	19.76	86.43	55.29	58.22	66.65	17.93

**Table 5:** Mean TNRs and the corresponding standard deviations are calculated across all evaluated sensor pairs.

	Euclidean Distance					Chebyshev Distance				
	TNR (%) for Sensor Pairs			Mean TNR (%)	Std. Dev. (%)	TNR (%) for Sensor Pairs			Mean TNR (%)	Std. Dev. (%)
k Values	11–12	6–10	1–2			11–12	6–10	1–2		
1	90.09	21.46	0.00	37.18	40.63	97.54	27.79	15.86	47.06	38.95
3	92.94	68.76	67.72	76.47	28.97	97.78	79.37	71.43	82.86	27.76
5	93.58	79.13	71.43	81.38	27.65	98.89	86.51	78.10	87.83	21.55
7	94.45	84.60	75.24	84.76	24.07	98.89	90.16	81.59	90.21	17.87
10	94.45	87.14	78.73	86.77	20.37	100.00	95.24	83.81	93.02	15.66
15	96.11	91.43	81.90	89.82	17.90	100.00	97.78	84.76	94.18	14.53
20	98.57	97.14	84.13	93.28	14.99	100.00	100.00	86.67	95.56	12.81
25	98.57	99.05	84.76	94.13	14.41	100.00	100.00	86.67	95.56	12.81
30	99.68	100.00	84.76	94.81	14.61	100.00	100.00	88.57	96.19	10.98
35	100.00	100.00	86.43	95.48	12.78	100.00	100.00	91.43	97.14	8.24

**Table 6:** Influence of the sensitivity factor x on the novelty detection performance using the Chebyshev distance metric and k = 10.

	Balanced Accuracy					True Negative Rate				
Sensitivity Factor x	11–12 (%)	6–10 (%)	1–2 (%)	Mean of Sensor Pairs (%)	Std. Dev. (%)	11–12 (%)	6–10 (%)	1–2 (%)	Mean of Sensor Pairs (%)	Std. Dev. (%)
0.00	86.25	62.86	70.87	73.33	16.70	72.50	31.93	52.86	52.43	30.88
0.50	93.02	68.77	77.58	79.79	17.29	86.05	48.23	72.31	68.86	31.13
1.00	96.23	72.53	75.94	81.57	17.70	92.47	66.01	77.14	78.54	26.23
1.50	97.34	72.38	71.40	80.37	18.87	94.68	73.99	77.46	82.05	25.02
2.00	98.31	71.84	69.37	79.84	20.19	97.22	79.52	79.05	85.27	23.50
2.50	98.65	70.03	68.50	79.06	20.36	98.89	84.13	81.59	88.20	20.18
3.00	98.02	69.41	67.21	78.21	20.34	98.89	91.11	83.49	91.16	16.89
3.25	98.28	69.00	65.82	77.70	20.50	100.00	94.29	83.81	92.70	15.84
3.50	97.94	67.97	64.92	76.94	20.77	100.00	95.56	83.81	93.12	15.61
3.75	97.56	68.15	63.32	76.35	20.84	100.00	97.46	83.81	93.76	15.44
4.00	97.24	67.55	62.50	75.76	21.04	100.00	98.73	83.81	94.18	15.45
4.50	96.31	65.41	61.16	74.29	20.84	100.00	99.68	84.13	94.60	15.22
5.00	95.17	63.89	59.89	72.99	20.51	100.00	100.00	84.76	94.92	14.64
5.50	93.77	62.44	57.99	71.40	20.46	100.00	100.00	84.76	94.92	14.64
6.00	92.53	61.48	57.14	70.38	20.30	100.00	100.00	84.76	94.92	14.64
6.50	90.90	61.18	56.59	69.56	19.60	100.00	100.00	86.03	95.34	13.42
7.00	88.69	60.37	55.85	68.30	18.65	100.00	100.00	86.67	95.56	12.81
7.50	87.44	59.51	55.35	67.44	18.07	100.00	100.00	87.62	95.87	11.90
8.00	85.52	59.14	55.12	66.60	17.21	100.00	100.00	88.57	96.19	10.98
8.50	83.79	58.64	54.34	65.59	16.50	100.00	100.00	88.57	96.19	10.98

**Figure A1:**
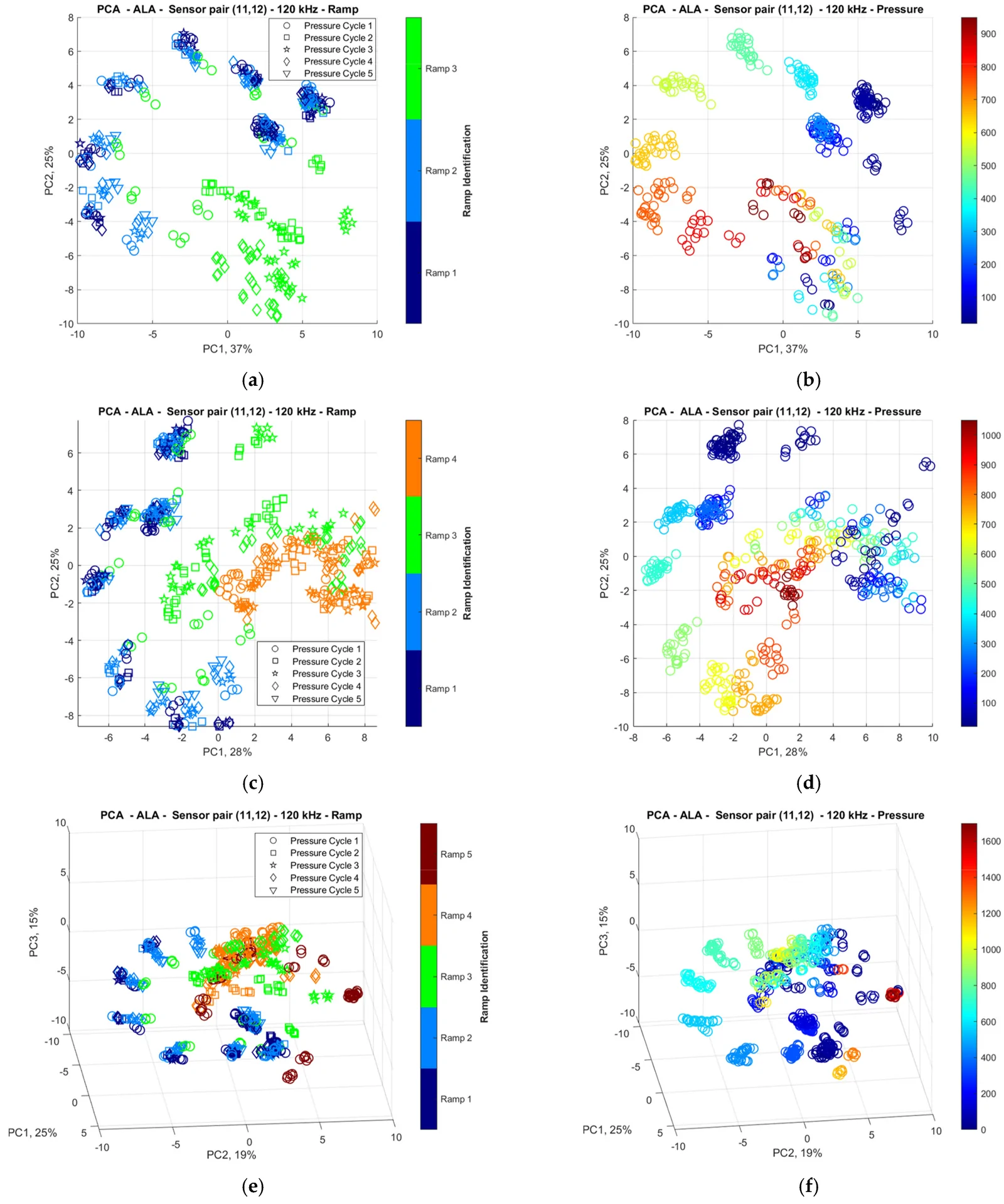
PCA projections of the extracted ALA features for sensor pair 11–12. The data points are colored according to the pressure ramps (**left**) and the applied pressure (**right**). (**a**,**b**) Ramps 1–3, (**c**,**d**) Ramps 1–4, and (**e**,**f**) Ramps 1–5 (burst).

**Figure A2:**
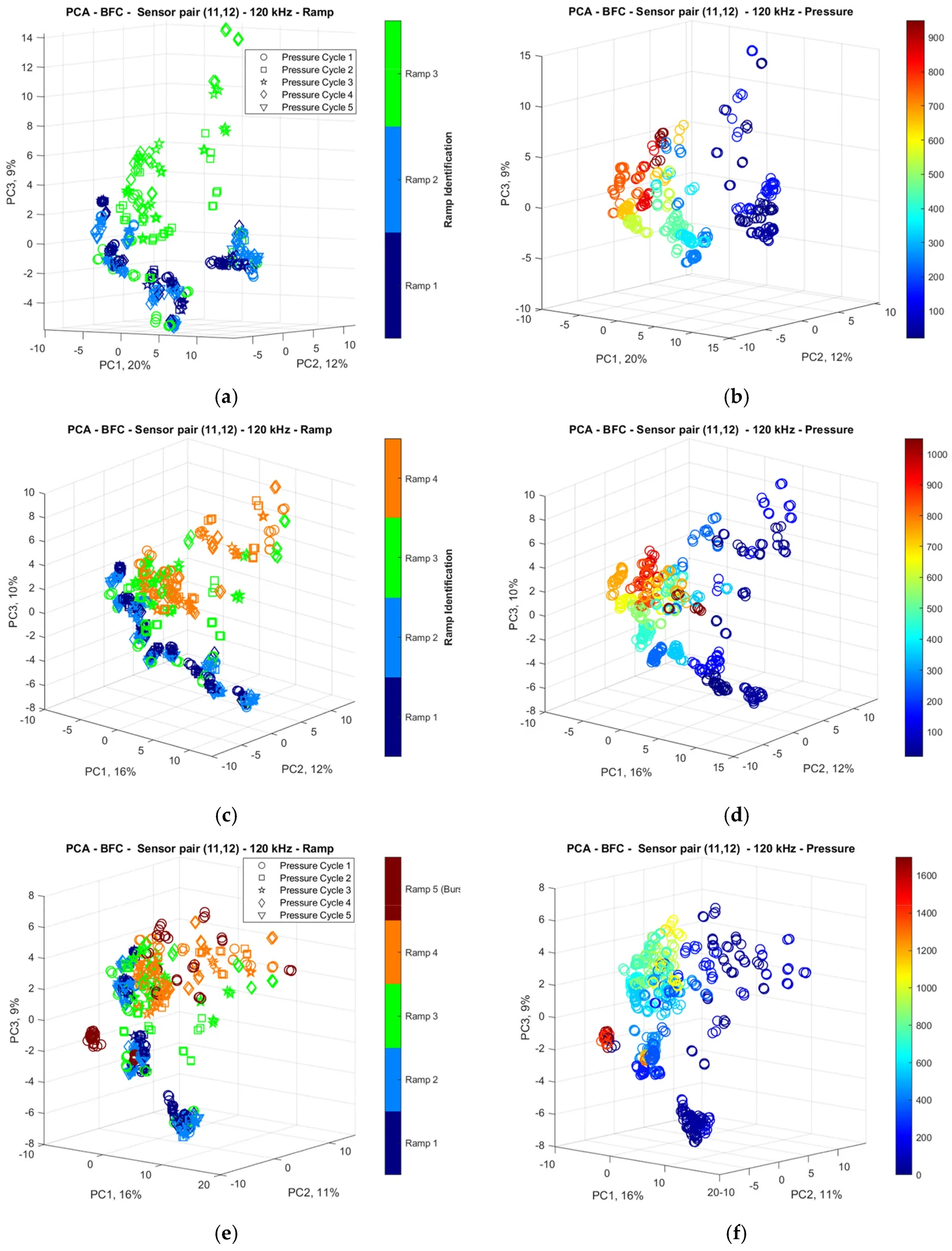
PCA projections of the extracted BFC features for sensor pair 11–12. The data points are colored according to the pressure ramps (**left**) and the applied pressure (**right**). (**a**,**b**) Ramps 1–3, (**c**,**d**) Ramps 1–4, and (**e**,**f**) Ramps 1–5 (burst).

**Figure A3:**
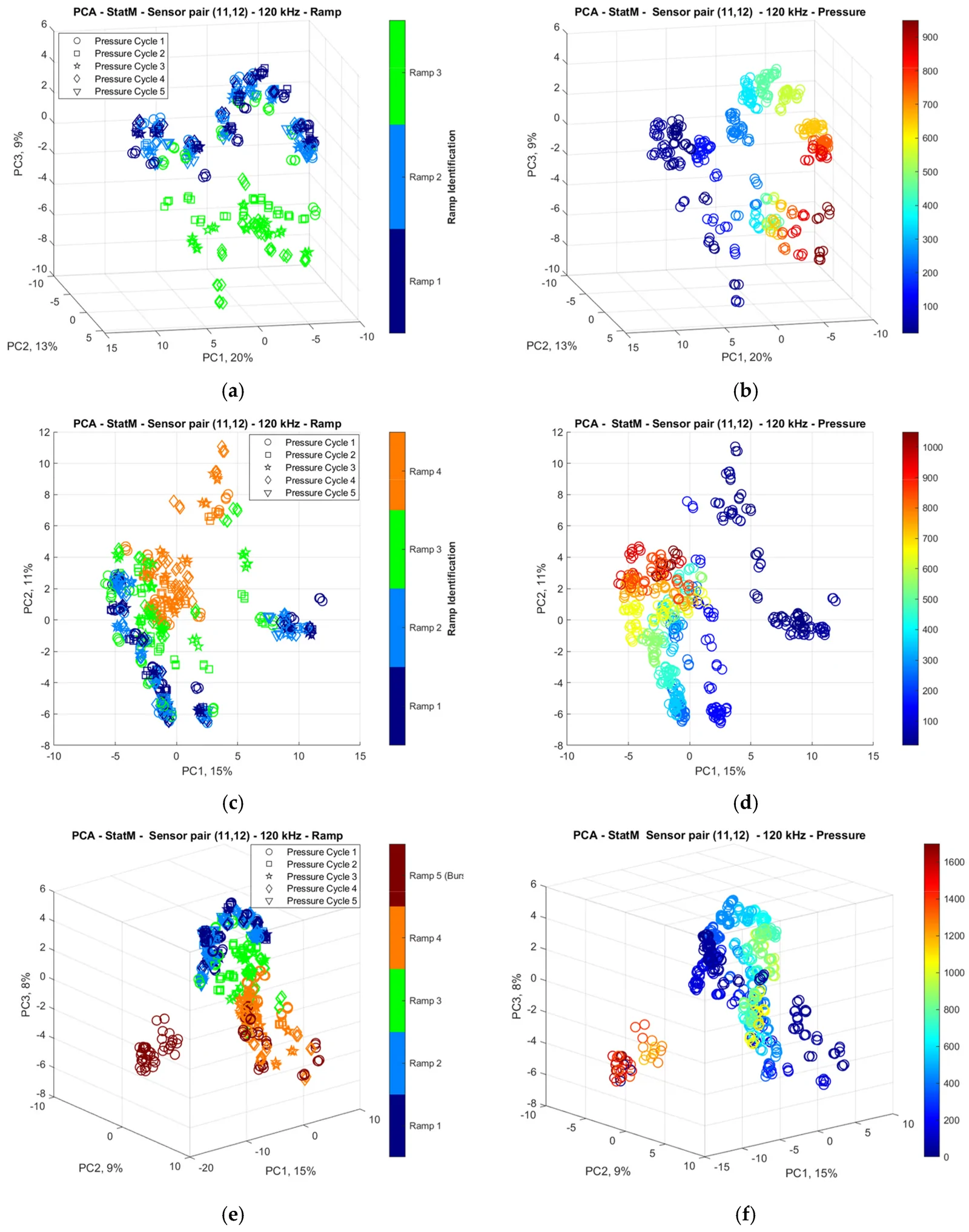
PCA projections of the extracted Statistical Moments features for sensor pair 11–12. The data points are colored according to the pressure ramps (**left**) and the applied pressure (**right**). (**a**,**b**) Ramps 1–3, (**c**,**d**) Ramps 1–4, and (**e**,**f**) Ramps 1–5 (burst).

**Figure A4:**
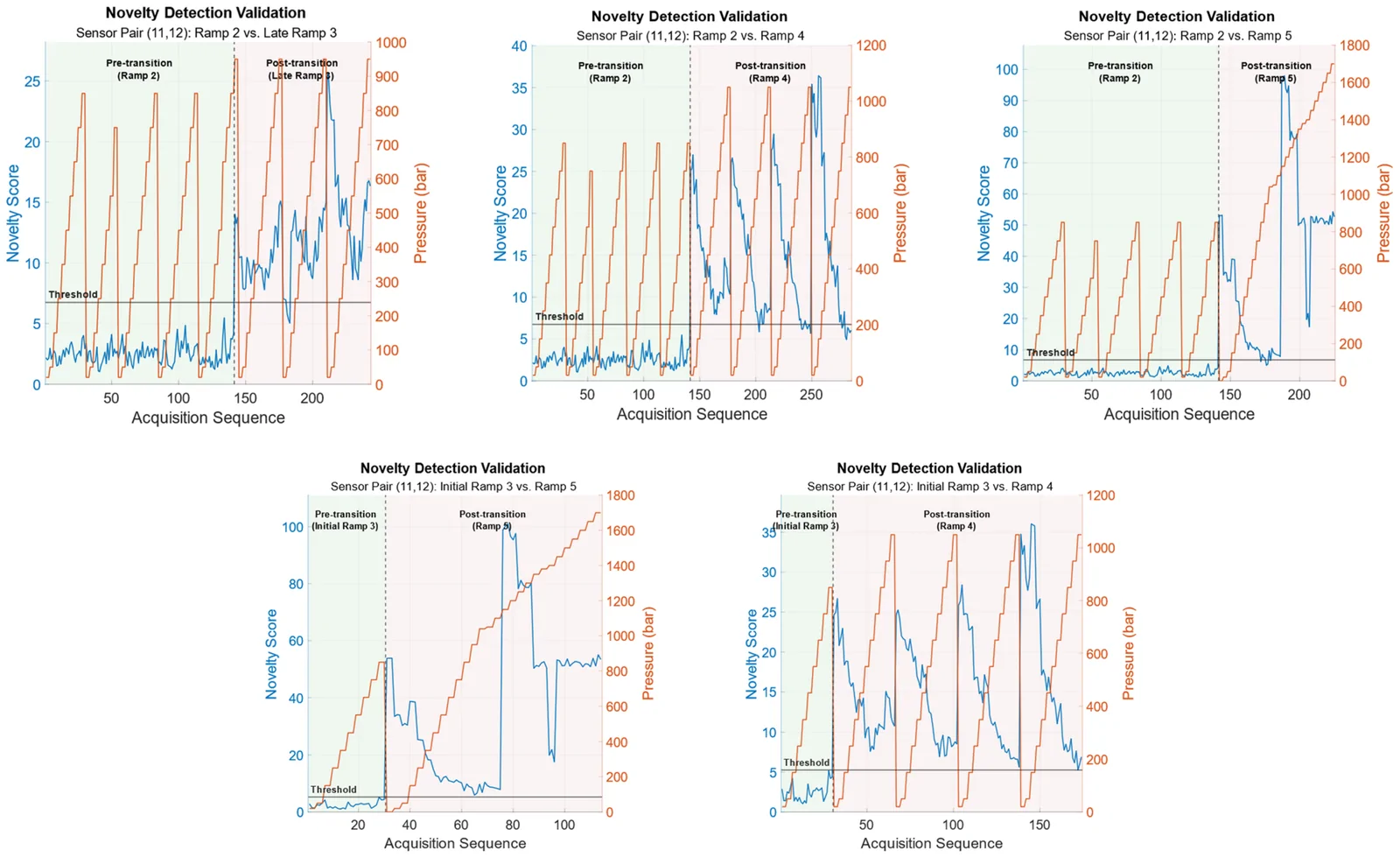
Remaining novelty detection validation results for sensor pair 11–12. The blue curve represents the novelty score, while the orange one represents the applied pressure. The vertical dashed line marks the beginning of the post-transition measurements.

**Figure A5:**
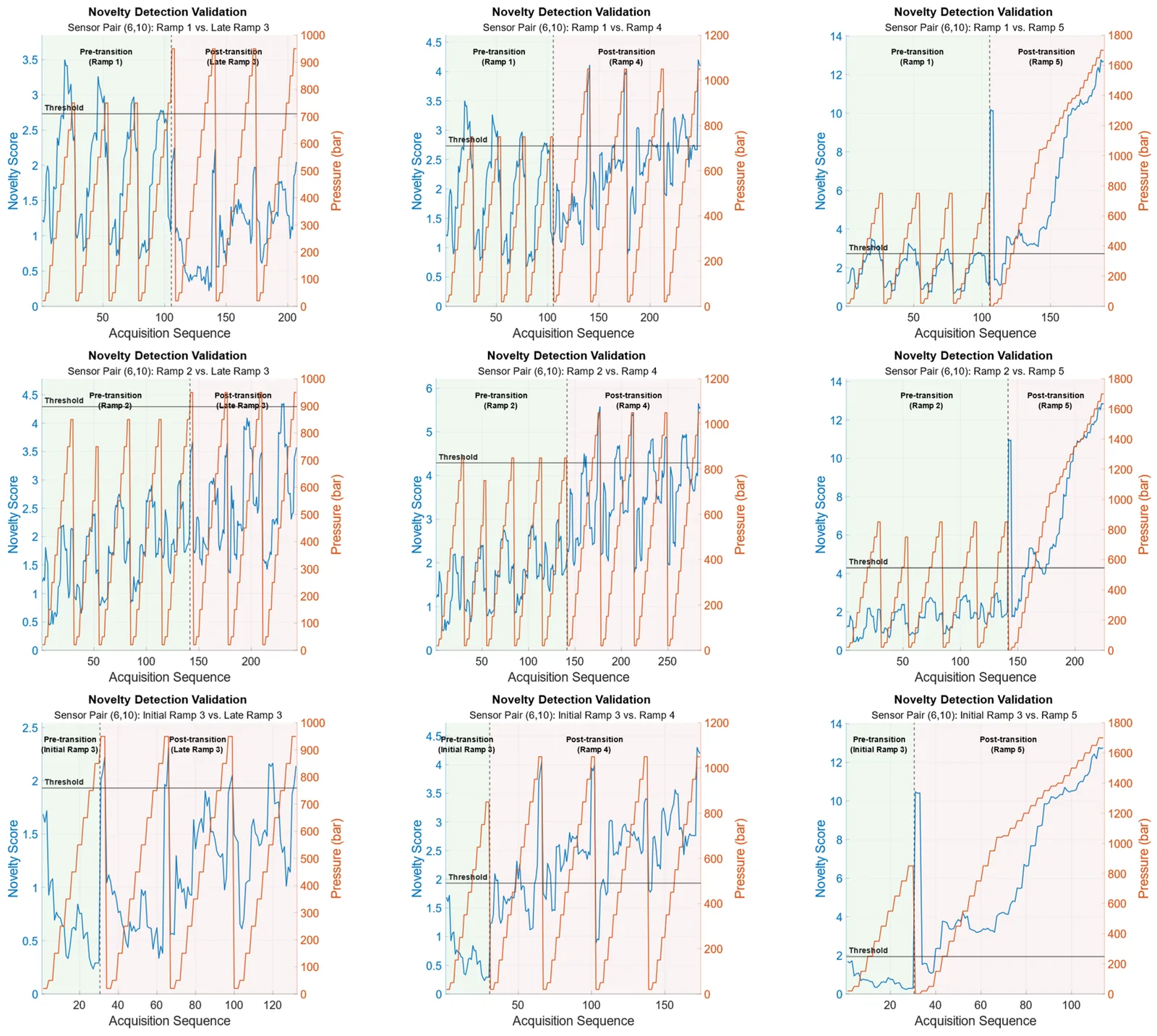
Novelty detection validation results for sensor pair 6–10. The blue curve represents the novelty score, while the orange curve represents the applied pressure. The vertical dashed line marks the beginning of the post-transition measurements.

**Figure A6:**
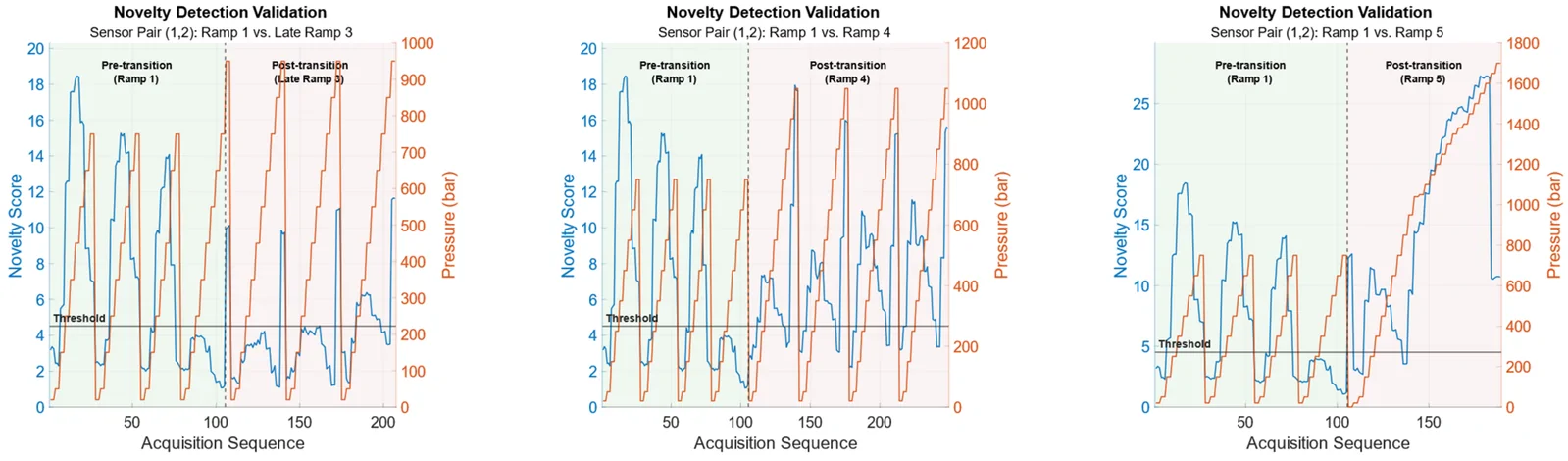

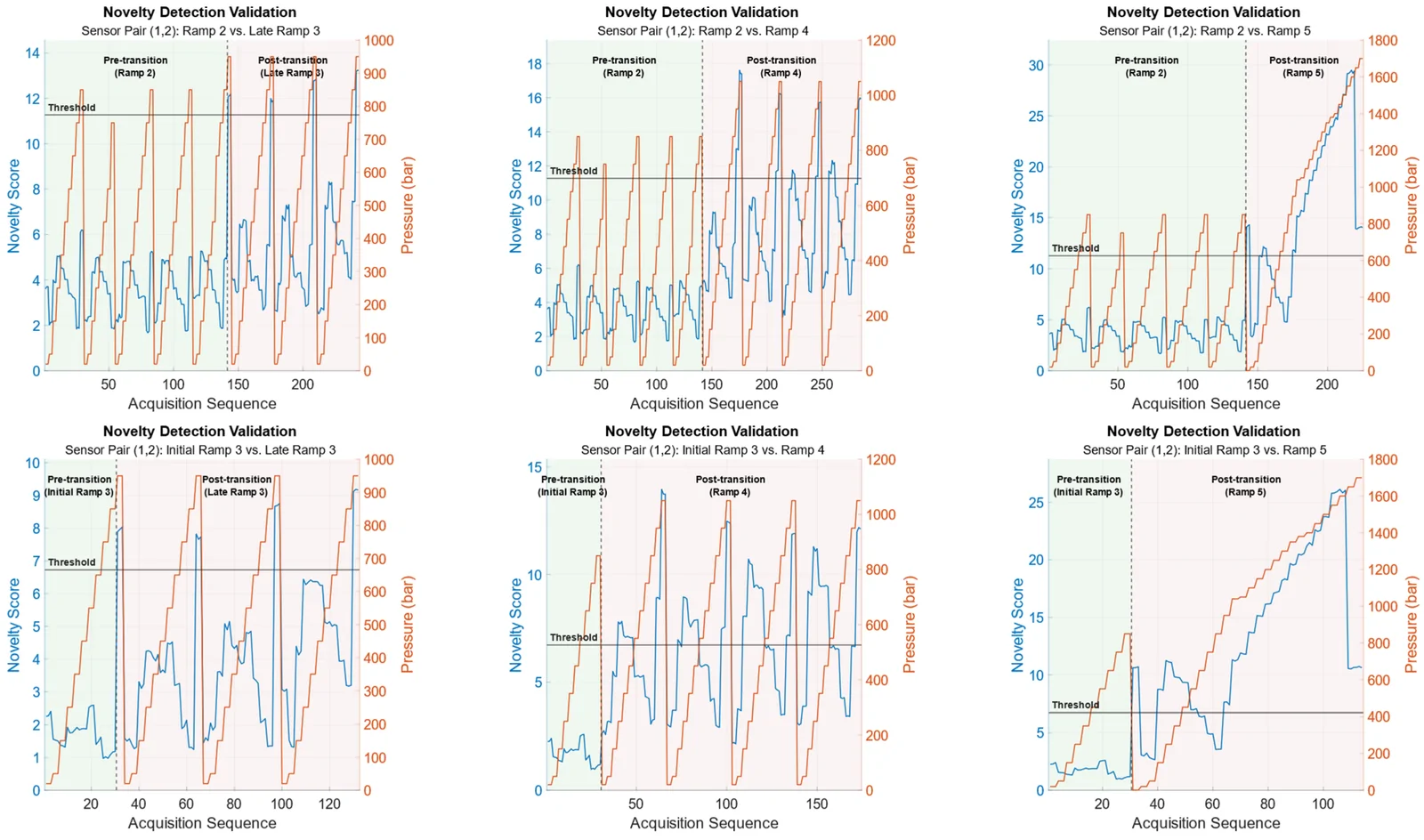
Novelty detection validation results for sensor pair 1–2. The blue curve represents the novelty score, while the orange curve represents the applied pressure. The vertical dashed line marks the beginning of the post-transition measurements.

## Data Availability

The raw data supporting the conclusions of this article will be made available by the authors on request.

## Abbreviations

The following abbreviations are used in this manuscript:

COPV	Composite overwrapped pressure vessels
SHM	Structural Health Monitoring
NDT	Non-Destructive Testing
ML	Machine Learning
UGW	Ultrasonic Guided Waves
kNN	k-Nearest Neighbors
BDW	Best Daubechies Wavelet
BFC	Best Fourier Coefficients
ALA	Adaptive Linear Approximation
BA	Balanced Accuracy
TPR	True Positive Rate
TNR	True Negative Rate
PC	Principal Component
PCA	Principal Component Analysis
LOGO-CV	Leave-One-Group-Out Cross-Validation

## Appendix B

### Appendix B.1

**Table A1:** Balanced accuracy (BA) for sensor pair 11–12 obtained with different feature extractors and parameter settings. The table rows are sorted in descending order of the mean BA over all validation folds.

Extractor	Parameter	Fold 1	Fold 2	Fold 3	Fold 4	Fold 5	Fold 6	Fold 7	Fold 8	Fold 9	BA (%)	Std. Dev. (%)
BDW	5	97.14	96.45	96.55	98.94	98.24	97.75	93.33	93.33	93.33	96.12	2.23
ALA	30	99.05	99.05	99.05	94.68	94.68	94.68	91.67	91.67	91.67	95.13	3.21
BDW	15	92.86	92.86	92.86	96.45	96.45	96.45	95.00	95.00	95.00	94.77	1.57
BDW	10	97.62	97.62	97.62	95.39	95.39	95.39	90.00	90.00	90.00	94.34	3.39
ALA	40	98.10	98.10	98.10	94.33	94.33	94.33	86.67	86.67	86.67	93.03	5.04
BDW	20	85.24	85.24	85.24	97.16	97.16	97.16	95.00	95.00	95.00	92.47	5.50
ALA	50	95.71	95.71	95.71	93.62	93.62	93.62	83.33	83.33	83.33	90.89	5.74
BFC	20	84.76	84.76	84.76	98.94	98.94	98.94	76.67	76.67	76.67	86.79	9.76
BFC	40	84.76	84.76	84.76	98.94	98.94	98.94	76.67	76.67	76.67	86.79	9.76
BFC	60	84.76	84.76	84.76	98.94	98.94	98.94	76.67	76.67	76.67	86.79	9.76
Statistical Moments	10	67.62	67.62	67.62	76.24	76.24	76.24	53.33	53.33	53.33	65.73	10.02
Statistical Moments	20	66.19	66.19	66.19	75.18	75.18	75.18	50.00	50.00	50.00	63.79	11.05
Statistical Moments	30	62.38	62.38	62.38	78.01	78.01	78.01	50.00	50.00	50.00	63.47	12.16
